# COG Complex Complexities: Detailed Characterization of a Complete Set of HEK293T Cells Lacking Individual COG Subunits

**DOI:** 10.3389/fcell.2016.00023

**Published:** 2016-03-30

**Authors:** Jessica Bailey Blackburn, Irina Pokrovskaya, Peter Fisher, Daniel Ungar, Vladimir V. Lupashin

**Affiliations:** ^1^Department of Physiology and Biophysics, University of Arkansas for Medical SciencesLittle Rock, AR, USA; ^2^Department of Biology, University of YorkYork, UK

**Keywords:** COG complex, Golgi apparatus, CRISPR, vesicle tethering, glycan processing, glycosylation, toxin trafficking

## Abstract

The Conserved Oligomeric Golgi complex is an evolutionarily conserved multisubunit tethering complex (MTC) that is crucial for intracellular membrane trafficking and Golgi homeostasis. The COG complex interacts with core vesicle docking and fusion machinery at the Golgi; however, its exact mechanism of action is still an enigma. Previous studies of COG complex were limited to the use of CDGII (Congenital disorders of glycosylation type II)-COG patient fibroblasts, siRNA mediated knockdowns, or protein relocalization approaches. In this study we have used the CRISPR approach to generate HEK293T knock-out (KO) cell lines missing individual COG subunits. These cell lines were characterized for glycosylation and trafficking defects, cell proliferation rates, stability of COG subunits, localization of Golgi markers, changes in Golgi structure, and *N*-glycan profiling. We found that all KO cell lines were uniformly deficient in *cis*/medial-Golgi glycosylation and each had nearly abolished binding of Cholera toxin. In addition, all cell lines showed defects in Golgi morphology, retrograde trafficking and sorting, sialylation and fucosylation, but severities varied according to the affected subunit. Lobe A and Cog6 subunit KOs displayed a more severely distorted Golgi structure, while Cog2, 3, 4, 5, and 7 knock outs had the most hypo glycosylated form of Lamp2. These results led us to conclude that every subunit is essential for COG complex function in Golgi trafficking, though to varying extents. We believe that this study and further analyses of these cells will help further elucidate the roles of individual COG subunits and bring a greater understanding to the class of MTCs as a whole.

## Introduction

Efficient docking and fusion of intracellular transport carriers in eukaryotic cells is tightly regulated by a family of multi-subunit tethering complexes (MTC) that sequentially and/or simultaneously interact with other components of vesicle docking and fusion machinery (Lupashin and Sztul, 2005; Yu and Hughson, 2010). The Conserved Oligomeric Golgi (COG) complex, the main MTC functioning at the Golgi, is a key player in intra-Golgi retrograde trafficking (Suvorova et al., 2002; Ungar et al., 2002; Miller and Ungar, 2012; Willett et al., 2013a,b). The COG complex is a hetero-octamer and a member of the CATCHR (complexes associated with tethering containing helical rods) family of proteins (Yu and Hughson, 2010). It is comprised of eight different protein subunits (named Cog1–8) (Whyte and Munro, 2001; Ungar et al., 2002) that are organized into two functionally distinct sub complexes or lobes: Cog1–4 in lobe A and Cog5–8 in lobe B (Fotso et al., 2005; Ungar et al., 2005). The two lobes are bridged together via an interaction between lobe A subunit Cog1 and lobe B subunit Cog8 (Fotso et al., 2005; Ungar et al., 2005). Previous studies on the interactome of the COG complex have revealed protein-protein interactions with several different families of trafficking regulatory proteins including SNAREs, SNARE-interacting proteins, Rab GTPases, coiled-coil tethers, and COPI subunits (Suvorova et al., 2002; Shestakova et al., 2006, 2007; Sohda et al., 2007, 2010; Sun et al., 2007; Miller et al., 2013; Willett et al., 2013a, 2014). Further studies revealed that the COG complex is required for the proper recycling of Golgi localized glycosylation enzymes (Kingsley et al., 1986; Podos et al., 1994; Shestakova et al., 2006; Steet and Kornfeld, 2006; Pokrovskaya et al., 2011; Cottam et al., 2014). In humans, mutations in COG complex subunits result in type II congenital disorders of glycosylation (CDG-II; Wu et al., 2004; Foulquier et al., 2006, 2007; Kranz et al., 2007; Paesold-Burda et al., 2009; Reynders et al., 2009; Ungar, 2009; Lübbehusen et al., 2010; Kodera et al., 2015). These COG complex deficiencies cause many developmental impairments including microcephaly, mental retardation, hypotonia, and issues with hemostasis (for review see Climer et al., 2015; Rymen et al., 2015). In addition to its roles in glycosylation and Golgi homeostasis, the COG complex is also hijacked by intercellular pathogens such as HIV and Chlamydia (Pokrovskaya et al., 2012; Liu et al., 2014). COG complex mutants have been found in a variety of model organisms including plants, fruit flies, worms, and yeast, but the phenotypes between these different model organisms vary greatly. In humans, COG complex function has been studied through case studies of CDGII-COG patients, patient fibroblasts in cell culture (which have limited proliferation potential, and may still have small amounts of mutant COG protein), or by using knock down and knock sideways assays in immortalized cell lines. Though these approaches have proved useful, they fail to allow for study of the long term effects of COG loss.

In the present study we report on a set of immortalized COG subunit KO cell lines made using CRISPR/Cas9 technology in HEK293T cells. These cells have allowed us to begin elucidating contributions of each subunit to overall complex function and stability, and also to probe the COG complexes' vast interactome for unique interacting partners and functions of the various subunits.

## Methods

### Reagents and antibodies

Reagents were as follows: GNL-Alexa 647 (Willett R. A. et al., 2013), RCAI-Rho (Vector Labs), CTxB-Alexa 647 (Molecular Probes), Subtilase cytotoxin (1.49 mg/ml; Paton et al., 2004). Antibodies used for immunofluorescence microscopy (IF) or western blotting (WB) were purchased through commercial sources, gifts from generous individual investigators, or generated by us. Antibodies (and their dilutions) were as follows: rabbit affinity purified anti-hCog3 (WB 1:10,000; Suvorova et al., 2001), hCog4 (WB 1:1000, this lab), hCog6 (WB 1:1000, this lab), hCog8 (WB 1:1000, this lab); mouse monoclonal anti-LAMP2 (WB 1:100, DSHB), anti-Cathepsin D (WB 1:500, Sigma), anti-GM130 (WB 1:1000, BD), and goat anti-GRP78 (WB goat 1:1000; Santa Cruz).

### Cell culture, transfection, and media collection

HEK293T cells (ATTC) were grown in DMEM/F12 medium (Thermo Scientific) supplemented with 10% FBS (Atlas Biologicals) and in the presence of antibiotic/antimycotics where noted. Cells were kept at 37° and 5% CO_2_ in a 90% humidified incubator. Cells were passaged via gentle resuspension.

HEK293T Cog1 through Cog8 stable knockouts were generated using CRISPR technique (Jinek et al., 2012; Cong et al., 2013; Mali et al., 2013). gRNA sequences were either provided by Horizon Discovery or purchased from Genecopoeia. From Horizon: Cog8 Guide ID:123842 Target: GGTGGAGGATGAAGGGCTCC, Cog1 Guide ID: 129696 Target: TTTCGAGACGCATGGAGCGG, Cog3 Guide ID: 113509 Target: GGC GCTGTTGCTGCTGCCTG, Cog4 Guide ID: 123995 Target: CGAATCAAGGTC CGCCATCT, Cog6 Guide ID: 113368 Target: GCTGCCAACGGCCTCAACAA. From Genecopia: Cog5 Catalog No. HCP200561-CG01-3-B-a Target: CAACTA GCAAAACTTGCCCA, Cog2 Catalog No. HCP205454-CG01-3-B-a Target: CTT GTTGATGAGTTCGACCA, Cog7 Catalog No. HCP222287-CG01-3-B-b Target: CCAAGAGGTGAACCACGCCG. HEK293T cells were transfected with plasmid containing gRNA with Cas9-dasherGFP (Horizon Discovery) or Cas9 with mCherry (Genecopia).

For transfection with Lipofectamine 2000 cells were plated on a 6-well plate (one well for each set of plasmids) to reach a confluency of 50–70% the next day (~1.5 × 10^6^ cells/well). Cells were placed in Optimem prior to transfection, the lipid-DNA complex was added to the cells and cells were allowed to incubate with the DNA overnight. The media was changed to regular culture media the following morning. Cells were checked at the 24 and 48-h time points to evaluate transfection efficiency (via fluorescence on the CRISPR/Cas9 plasmid).

To obtain conditioned media from the cells to analyze cell secretions, cells went through the following protocol: Media was removed then cells were washed with PBS 3x. Serum free, chemically defined media (BioWhittaker Pro293a-CDM, Lonza) supplemented with glutamine was then added to the cells and the cells were incubated at 37°C and in 5% CO_2_ for 48 h before media was collected. Media was then spun at 3000 g to remove any cells or cell debris. The supernatant was removed and stored at −20°C until analyzed by SDS-PAGE and western blot.

### Immunofluorescence microscopy

Cells were grown on 12 mm glass coverslips (#1, 0.17 mm thickness) 1 day before transfection. After transfection cells were fixed and stained as described previously (Pokrovskaya et al., 2011). In short, cells were fixed in 4% paraformaldehyde (16% stock solution diluted in Dulbecco's phosphate-buffered saline (DPBS); Electron Microscopy Sciences). Cells were then treated with 0.1% Triton X-100 for 1 min and with 50 mM ammonium chloride for 5 min. Cells were washed with DPBS and blocked twice for 10 min with 1% BSA, 0.1% saponin in DPBS. Cells then were incubated for 1 h with primary antibody diluted in antibody buffer (1% cold fish gelatin, 0.1% saponin in DPBS) at room temperature. Cells were washed four times with DPBS and incubated for 30 min with fluorescently tagged secondary antibody in antibody buffer at room temperature. Coverslips were washed four times with DPBS, rinsed with ddH2O, and mounted on glass microscope slides using Prolong®; Gold antifade reagent (Life Technologies). Cells were imaged with the 63X oil 1.4 numerical aperture (NA) objective of a LSM510 Zeiss Laser inverted microscope outfitted with confocal optics. Image acquisition was controlled with LSM510 software (Release Version 4.0 SP1). Processing was done using this software as well as ImageJ.

### Lectin staining and immunofluorescence

Lectin staining was performed as previously described (Willett R. A. et al., 2013) with minor modifications. Cells were seeded on to collagen coated glass. When cells were 75–100% confluent coverslips were removed and washed with warm PBS then fixed in 1% paraformaldehyde in PBS for 15 min followed by a wash and a 10-min block with 1% BSA in PBS. The blocking step was repeated, then cells were incubated with the lectin diluted 1:1000 in 1% BSA solution for 30–60 min. Cells were washed 5 times and fixed for 15 min with 4% paraformaldehyde in DPBS for intracellular staining. The above protocol for traditional immunofluorescence to look at intracellular proteins was followed with an optional urea treatment (2–3 min) prior to blocking for better antibody binding.

### Flow cytometry

Cells were grown to 80–100% confluency then resuspened in ice cold 0.1% BSA by gentle pipetting and placed in an Eppendorf tube. Cells were pelleted at a low speed (600 g for 3 min) and resuspended in 0.1% BSA containing the lectin of choice (GNL, Ricin, or Cholera toxin) at a 1:1000 dilution. Cells were incubated in lectin solution on ice for 30–60 min. Cells were then spun down and resuspended in ice cold 0.1% BSA (plus DAPI for viability gaiting) by gentle pipetting. Cells were analyzed using the NxT Attune flow cytometer. Cells were gated for live cells (DAPI excluding cells), singlets, then for correct cell size vs. complexity. Analysis was done using FlowJo software.

### Cell sorting and colony expanding

For cell sorting, lectin staining was done as detailed above. Cells were spun down at a 600 g then resuspended in cell sorting media [PBS, 25 mM Hepes pH = 7.0, 2% FBS (heat inactivated) 1 mM EDTA, 0.2 μm sterile filtered] and filtered through a filtered cap 5 ml- 12 × 75 mm polystyrene round bottom tube before single cell sorting. Sorting was based on high-GNL-Alexa 647 fluorescence into a 96-well plate containing culture medium plus antibiotic/antimycotics. Cell sorting was done using FACSARIA at the UAMS Flow Cytometry core facility.

The 96-well plates were analyzed for colonies 10–14 days after sorting. Wells with colonies were marked and allowed to grow for 1 week more before expanding. After 21 days, colonies were expanded from 96-well to 12-well plates via resuspension. Cells were maintained in antibiotic/antimycotic media and allowed to grow further. Once colonies were split onto 10 cm dishes (around 4–6 weeks after initial transfection) aliquots were cryopreserved in freezing media (90% FBS plus 10% DMSO) and the remaining cells were used for other analysis, including sequencing, western blot, and flow analysis.

### Chromosomal DNA Prep, PCR, and sequencing

For chromosomal DNA prep cells were resuspended in culture media and an aliquot was taken for counting. After cell density was determined 100,000 cells were removed and placed into a microcentrifuge tube. Cells were spun down at low speed (600 g for 3 min) and resuspended in PBS then spun down again. 0.5 mL of Quick Extract DNA extraction solution was added then cells were resuspended via vortex. Cells were placed at 63°C for 8 min, vortexed and placed at 98°C for 2 min per the manufacturer's instructions. Chromosomal DNA was stored at −20°C until use.

PCR using chromosomal DNA as a template was done for a 300–800 base pair region surrounding the CRISPR cut site. Two 50 μL reactions were done for each potential knock out cell line as well as two reactions of a wild type control for each. Each reaction contained the following: 2 mM each of targeted COG subunit forward and reverse primer, 2–3 μL of the chromosomal DNA, 2.5 μL of DMSO, 4 μL of dNTPs, 5 μL of 5x exTaq buffer, and 0.2 μL of exTaq polymerase. Reactions were run with the following settings:

1. 95°C, 1:00 min
2. 95°C, 0:10
3. 57°C, 0:10
4. 72°C, 0:30
5. Repeat 2–4, 35x
6. 6 72°C, 0:30
7. 4°C, ∞

Reaction products were then gel purified using a Gel Extraction kit (Zymo Reaserch) and following the manufactures protocols. Samples were then sent for sequencing at the UAMS DNA Sequencing core facility.

### Subtilase cytotoxin assay

Subtilase cytotoxin (SubAB) assay was essentially performed as in Smith et al. (2009) with minor modifications. Both control and COG KO HEK293T cells were grown on 12-well culture plates to 70–80% of confluency. Cells were placed in 10% FBS in DMEM/F-12 (50:50) without antibiotics and antimycotics for at least 1 h before the assay was performed. The subtilase cytotoxin was diluted in the same medium (without antibiotic) and warmed up to 37°C. The time course is 0-, 20-, 40-, 60- 120-, and 180-min. Cells were incubated with the subtilase cytotoxin (0.05 μg/ml for the appropriate times in the 37°C incubator with 5% CO_2_). Cells were then washed off with PBS and lysed with 2% SDS warmed up to 95°C. Cell lysates, 10 μl of each, were loaded on a 4–15% gradient gel and immunoblotted with anti-GRP78 (Santa Cruz; C-20) antibody. All experiments were performed in triplicates. The blots were scanned and analyzed with an Odyssey Infrared Imaging System (LI-COR, Lincoln, NE).

### High pressure freezing, freeze substitution, and electron microscopy

Sapphire disks were carbon coated then collagen coated (Corning) per the manufacturer's instructions. Disks were then sterilized under UV light and transferred to new sterile 3 cm dishes (one dish with 3 disks per sample), cells were plated on top and allowed to grow until a confluency of 80–100% was reached on the disks. The media was then changed for each of the plates to fresh culture media and cells were allowed to incubate at 37°C for 2–3 h before high pressure freezing. Disks containing cells were high pressure frozen in cryoprotectant (PBS with 2% agarose, 100 mM D-mannitol, and 2% FBS) using a Leica EM PACT2 high pressure freezer with rapid transfer system, then transferred under liquid nitrogen into tubes containing a staining cocktail (acetone with 2% osmium tetroxide, 0.1% Glutaraldehyde, and 1% ddH_2_O) pre frozen in liquid nitrogen as well. Tubes were then transferred to a freeze substitution chamber at −90°C on the following schedule: −90°C for 22 h, warm 3°C/h to −60°C, −60°C for 8 h, warm 3°C/h to −30°C, −30°C for 8 h, warm 3°C/h to 0°C.

Sample tubes were then placed on ice and washed with acetone 3x. Samples were than stained with a 1% Tannic acid/1% ddH_2_O solution in acetone on ice for 1 h before three more acetone washes. Samples were than stained with a 1% osmium tetroxide/1% ddH_2_O solution in acetone on ice for 1 h then washed three more times in acetone before the embedding process.

Samples were embedded in Araldite 502/Embed 812 resin (EMS) with DMP-30 activator added in a Biowave at 70°C under vacuum for 3 min for each embedding step. Samples were then baked at 60°C for 48 h before sectioning. Samples were stained post sectioning with uranyl acetate and lead citrate before imaging. Ultrathin sections were imaged at 80 kV on a FEI Technai G2 TF20 transmission electron microscope and images were acquired with a FEI Eagle 4kX USB Digital Camera.

### Cell lysis and western blot

Media was removed from one well of a 6-well plate (cells at ~90% confluency) for each cell line and cells were washed gently with PBS. Cells were resuspended in PBS via gentle resuspension and placed into a microcentrifuge tube. Cells were then pelleted at 600 g for 3 min. PBS was removed and 250 μL of 95°C 2% SDS was added to lyse cells. The mixture was vortexed and heated at 95°C for 5 min. Fifty microliters of 6x sample buffer containing 5% β-mercaptoethanol was added and vortexed. Samples were stored at −20°C until use.

For western blot analysis 10–15 μL lysates were added to wells a 4–15% gradient gel. The gel was then run at 180 V until the dye reached the bottom. Proteins were transferred to a nitrocellulose membrane. The membrane was stained with Ponceau S Stain and a picture was taken. The membrane was then washed and blocked for 20 min. Primary antibodies in blocking buffer were added and the membrane was incubated overnight at 4°C. The following day the membrane was washed 3x and incubated with secondary antibody in 5% evaporated milk in PBS for 40 min. Membrane was washed 4x and imaged using the Odyssey imaging system. Analysis was done in Licor Image Studio light.

### Glycan sample preparation and mass spectrometry

Procedure was performed as in Abdul Rahman et al. (2014) with some modifications.

In brief, cells were grown to 80–90% confluency. Media was removed and cells were washed 5x with PBS. Cells were then resuspended in PBS via gentle pipetting and placed on ice. Cells were then spun down at 14,000 g for 5 min. PBS was then removed and more was added. Cells were spun down again then the supernatant was removed. Glycan lysis buffer (4% (w/v) SDS, 100 mM Tris/HCl pH 7.6, 0.1 M dithiothreitol) was added in a 1:10 pellet/lysis buffer volume ratio. The sample was heated to 95°C for 5 min. The lysate was centrifuged at 14,000 g for 10 min, and the supernatant was collected and kept at −80°C.

Filter-aided *N*-glycan separation (FANGS) was performed as described (Abdul Rahman et al., 2014). Specifically, following dilution in urea buffer (8 M urea, 100 mM Tris/Cl, pH 8.5) the lysate was transferred into a 30 kDa ultrafiltration tube (Millipore). Subsequently repeated centrifugations and dilutions with urea buffer were performed interspersed with an iodoacetamide treatment (40 mM in 300 μL urea buffer). Finally, the membrane filter was washed three times with ammonium bicarbonate (50 mM, pH 8.0), and the sample treated with 8 units of PNGase F in 100 μL ammonium bicarbonate buffer for 16 h at 37°C. Glycans were eluted from the filter by centrifugation followed by a wash in HPLC grade water. Released *N*-glycans were dried and permethylated in alkaline DMSO using iodomethane and dried as described (Abdul Rahman et al., 2014). Permethylated *N*-glycans were dissolved in 20 μL of methanol, 2 μL of this solution was then mixed with 2 μL of 20 mg/mL 2,5-dihydroxybenzoic acid (DHB) in 70% methanol and 1 μL of sodium nitrate (0.5 M), spotting 2 μL of this onto a MALDI target plate. Mass spectra were acquired on a 9 T solariX FTICR mass spectrometer (Bruker Daltonics) recorded over the m/z range 400–4000 in positive ion mode with 500 laser shots. Eight scans were averaged and the laser power was set between 30 and 40%. Spectra were calibrated externally using a Bruker Peptide Mix II. *N*-glycans were identified by their accurate mass and isotope pattern, and included in the analysis if at least two isotope peaks were above a signal to noise ratio of three. Glycan intensities were calculated by summing the peak area divided by the half maximal width for each isotope. Relative quantification was achieved by normalizing glycan intensities within each spectrum to the sum of intensities for the glycan species observed in all analyzed cell lines. Relative abundances were averaged from biological replicates and error bars indicate the standard error of the mean.

## Results

### Cells deficient in COG complex subunits express an increased amount of terminal high mannose residues on the plasma membrane

*N*-glycosylation begins in the ER where the forming glycan chains are added to nascent proteins. The glycoprotein is then modified and transferred to the Golgi where the oligomannose type glycans, which contain terminal mannose residues, are then trimmed and processed. This process, which continues as the protein progresses through the Golgi, leads to hybrid then finally mature, complex oligosaccharides (Stanley, 2011).

Our group has previously shown that COG complex subunit knock downs (KD) in HeLa and HEK293T cells cause altered binding of several lectins due to impaired glycosylation of plasma membrane glycoconjugates while in the Golgi (Shestakova et al., 2006; Richardson et al., 2009; Pokrovskaya et al., 2011; Willett R. A. et al., 2013; Ha et al., 2014). We used this knowledge to screen HEK293T cells subjected to CRISPR/Cas9–based gene editing to isolate COG KOs. For this preliminary screen we chose *Galanthus nivalus* lectin conjugated with Alexa 647 (GNL-647) as a tool for selection. GNL binds to terminal α1-3 linked mannose residues (Shibuya et al., 1988) to all tested COG KD cells (Pokrovskaya et al., 2011) making it a helpful probe for immature glycans. By treating non-permeabilized cells with fluorescently tagged GNL, only immature glycans on the cell surface bind the lectin, making cells with glycosylation defects easy to sort from the transfected population.

**Figure 1:**
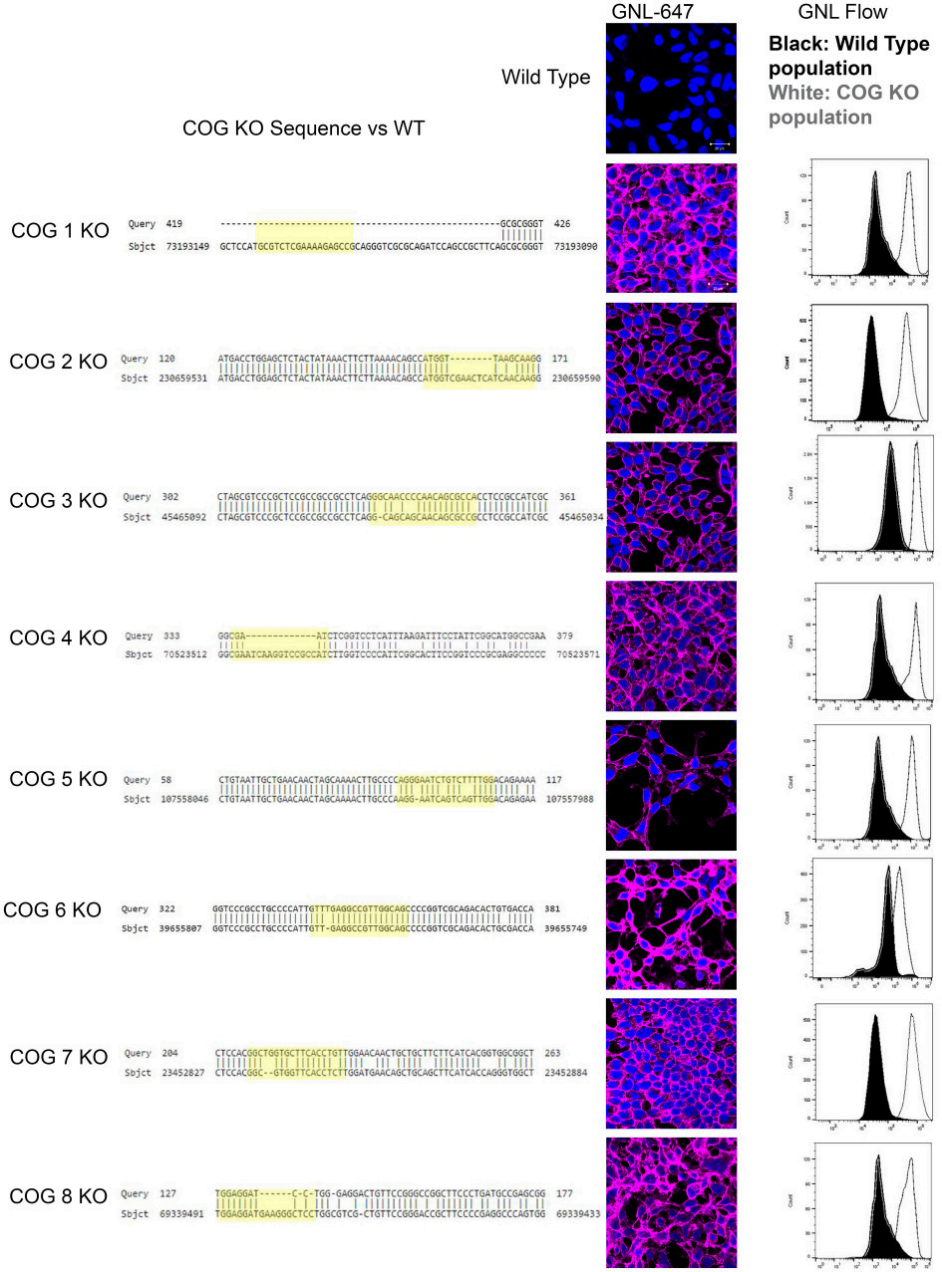
**COG KO validation**. **Left column**: Sequence alignment of mutant and control DNA. Chromosomal DNA was amplified at the CRISPR target region by high fidelity PCR. The expected cut site based on the guide RNA is highlighted in yellow. The central column shows plasma membrane staining of WT HEK293T and COG KO cells with *Galanthus nivalis* lectin (GNL-pink). Nuclei stained with DAPI (blue). **Right column**: cells were analyzed using flow cytometry for GNL staining (wild-type cells are in black, COG KO cells are in white).

**Figure 2:**
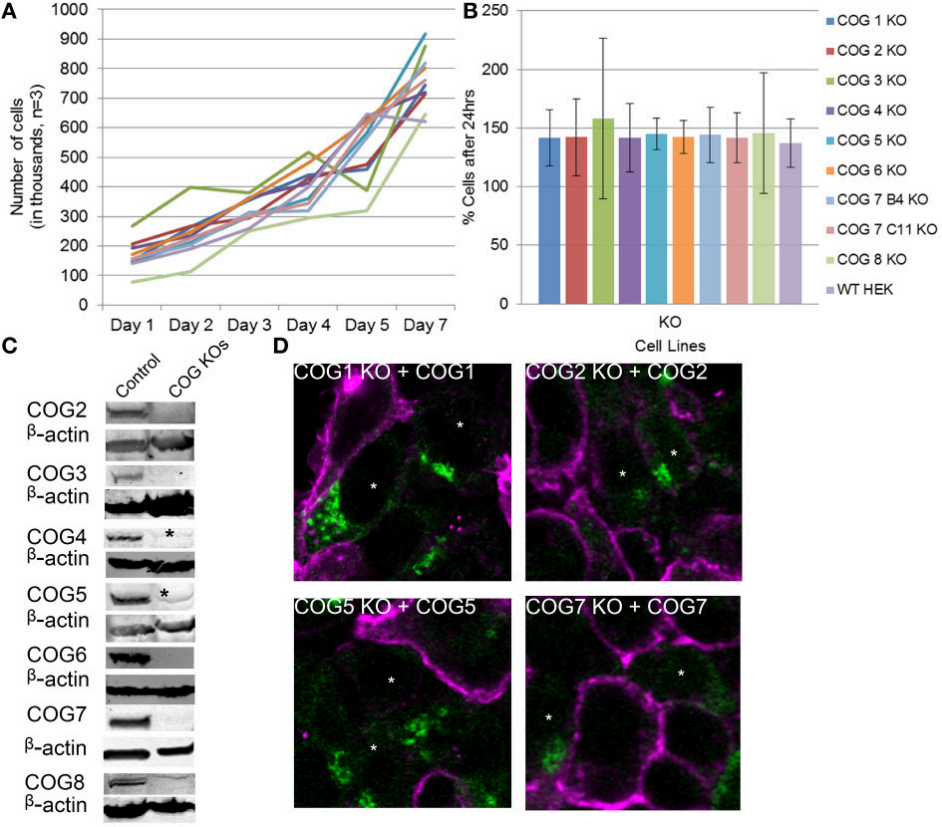
**Growth and rescue of COG KO cells**. **(A)** Growth of WT and KO cells. Cells were plated in 24 well plates in triplicate at 100,000 cells per well (Day 0). Cells were counted at the indicated time points over a week and cell counts were plotted. **(B)** The average growth in a 24 h period was calculated by (# of cells on day n/ # of cells on day n-1)^*^100 to get percent growth per day. Growth percentages over the week for each cell line were averaged. **(C)** Western blot analysis for each COG subunit KO cell line. β-actin is used as a loading control. Asterisks indicate non-specific bands. **(D)** Rescue of COG dependent glycosylation defect. Missing COG subunits (green) were transfected into KO cells. Seventy two hours later cells were fixed and stained with GNL-Alexa 647 (pink). Note that GNL binding was significantly reduced in cells expressing COG subunits.

Preliminary analysis revealed that 8 days after transfection with individual COG-subunit-specific CRISPR constructs a subpopulation of cells (around 5% of the total population) appeared that have high GNL binding compared to control cells (data not shown). From the 5% GNL positive population observed by flow cytometry, presumed COG KO cells were single cell sorted into a 96 well plate. Each plate yielded ~10–15 individual colonies. On the secondary GNL binding test several colonies demonstrated diminished GNL staining (~3 for each plate) and these clones were always still positive for the targeted subunit and served as an internal control. We preserved at least 2–5 Cog negative clones for each subunit KO as assessed by high GNL binding (assessed by IF, Figure 1). For further confirmation of COG KO induced high GNL binding, flow analyses were performed on these clones. KO cells labeled with GNL-647 revealed a uniform, bright plasma membrane staining that was distinct from control HEK293T cells (Figure 1). This increased amount of plasma membrane glycoconjugates with terminal α1-3 linked mannose residues indicates altered activities in *cis*-Golgi Mannosidase I enzymes (MAN1A1, MAN1A2, MAN1C1) as well as in *cis*/medial-Golgi localized Mannosidase II (MAN2A) and/or GlcNAc-T1 (MGAT1) transferase that were shown previously to be COG complex dependent (Pokrovskaya et al., 2011). From this we concluded that GNL binding is a powerful selection strategy for phenotypic sorting of all COG subunits KOs. Clonal populations of COG KOs were further validated by sequencing and western blot (Figures 1, 2). Because all CRISPR gRNA sequences were designed to target the first exon of COG genes, we have amplified the first exon from chromosomal DNA extracted from both control and KO cells with high-fidelity PCR. Sequencing of resulted PCR products revealed significant deletions and/or mutations in the first exon of each of targeted genes (Figure 1). We also confirmed a complete loss of corresponding COG subunits by Western blot (WB) for Cog2–8 KOs (Figure 2C).

Because antibodies for Cog1 are currently not available for western blot, we next sought to further validate this cell line and others by rescuing the glycosylation defects by transient expression of the myc-tagged knocked-out COG subunit (Figure 2D). Four days after transfection, each replacement COG subunit was observed on the Golgi in cells receiving the plasmids. These cells also showed WT (decreased) levels of GNL-647 binding to plasma membrane in contrast to their untransfected neighbors (Figure 2D). This rescue further validated the COG KO cell lines and supports the idea that cis/medial-Golgi glycosylation is dependent on the entire COG complex and that this is not an off target effect of our CRISPR protocol.

To further characterize the COG KO cell lines and test if aberrant glycosylation or impairment of COG-dependent interactions affected cell growth, cell proliferation was tracked (Figures 2A,B). Surprisingly cell lines showed no change from wild-type HEK293T cells in proliferation rates indicating that, in HEK293T cells, every COG complex subunit is not essential for cell growth and division.

**Figure 3:**
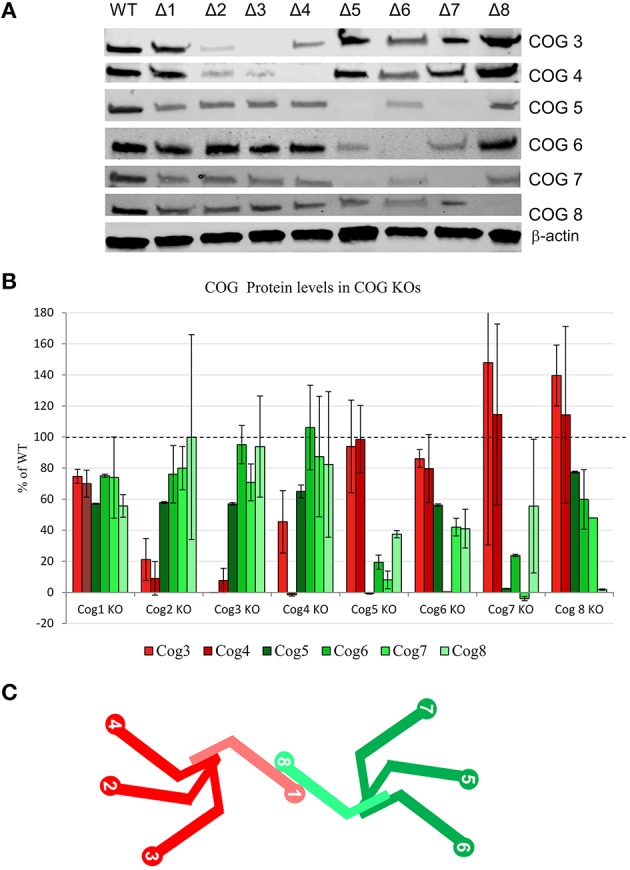
**COG protein stability upon loss of other COG proteins**. **(A)** All COG KO cells were probed by western blot for Cog3, 4, 5, 6, 7, and 8 protein levels. β-actin used as a loading control. **(B)** Quantification of 2 separate western blot analyses. Each Cog protein amount was first standardized to β-actin then compared to WT levels. **(C)** A model of COG subunit interactions. lobe A is shown in shades of red and lobe B is shown in shades of green. Cog1 and Cog8 are lighter in color to show that they also gain stability from one another in addition to their lobe partners.

To probe for the stability of remaining COG subunits in the absence of individual subunits, lysates of KO and WT cells were separated on SDS-PAGE and probed for antibodies to Cog3, 4, 5, 6, 7, and 8 (Figure 3A). (We were not able to include Cog1 due to lack of working antibodies. Cog2 was also omitted from this assay due to lack of sufficient amounts of this antibody to perform quantification). We have found that Cog3 and 4 protein levels were drastically impacted in Cog2, 3, and 4 KO cells indicating that these subunits are only stable in the context of lobe A subcomplex. Similar situation was observed for lobe B. Cog6 protein level was reduced in Cog5, 6, and 7 KO (Figure 3B). Interestingly Cog5 and 7 protein levels appear to be decreased to < 10% when the other protein is knocked out confirming our previous conclusion that interaction between these two partners is essential for their stability (Ha et al., 2014). Cog8 protein levels were reduced upon lobe partner loss but to a far less extent, indicating that Cog8 gains stability from other partners as well, likely from its interaction with Cog1. Cog1 interacts with Cog8, and this interaction is responsible for bridging the two lobes (Oka et al., 2005; Ungar et al., 2005). Cog proteins in opposing lobe KOs were occasionally reduced, but the reduction was never more than 45%, further supporting the bi-lobed model. We have summarized the supposed subunit interaction based on these assays in Figure 3C.

### Cells deficient in the COG complex subunits have an altered golgi morphology

We next investigated changes in Golgi structure in cells deficient for individual COG subunits. It has been previously shown that Golgi morphology was altered in both fibroblasts from COG-CDG patients (Reynders et al., 2009; Kudlyk et al., 2013) and in HeLa cells treated with siRNA to COG subunits (Zolov and Lupashin, 2005; Shestakova et al., 2006). The defects associated with the Golgi morphology were most notable in lobe A KDs.

**Figure 4:**
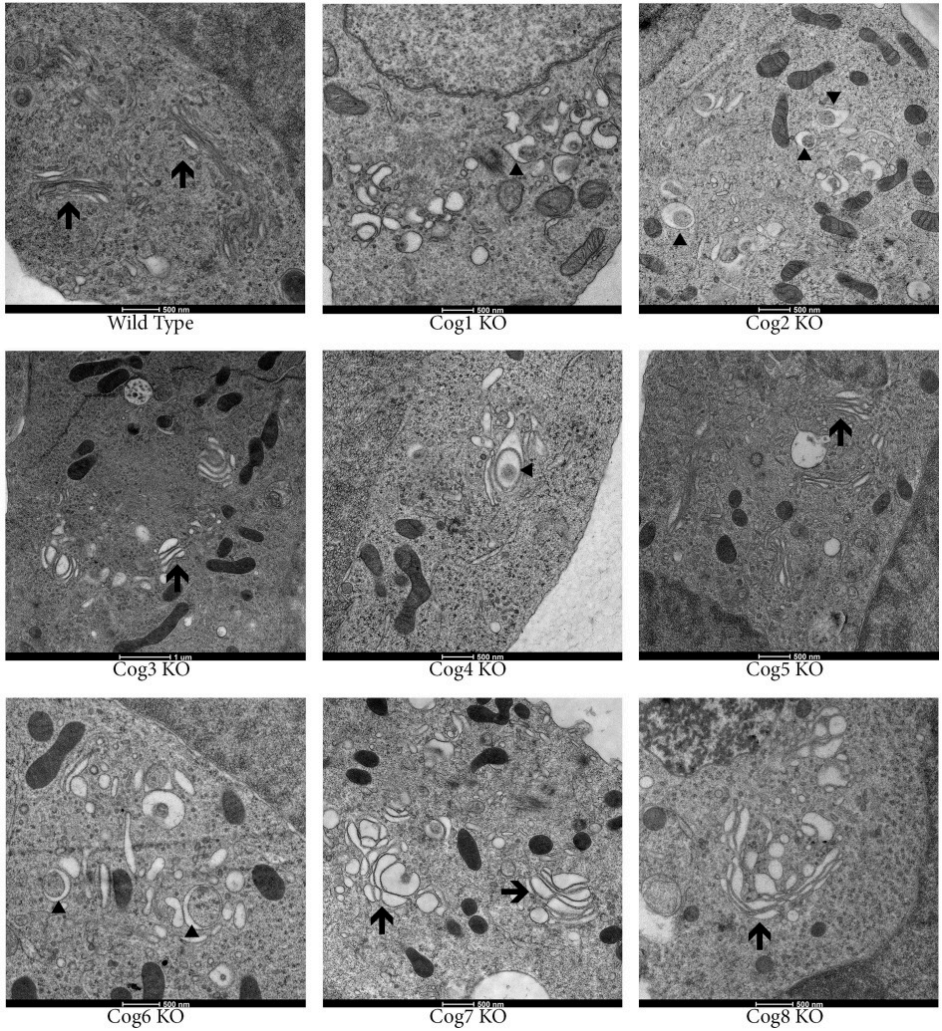
**Golgi structure is severely distorted in COG KO cells**. Cells were grown on carbon and collagen coated sapphire disk then subjected to high pressure freezing and freeze substitution. Samples were stained with tannic acid and osmium before embedding then uranyl acetate and lead citrate staining post sectioning. Arrows indicate Golgi stacks. Arrowheads indicate autophagosomal like structures.

**Figure 5:**
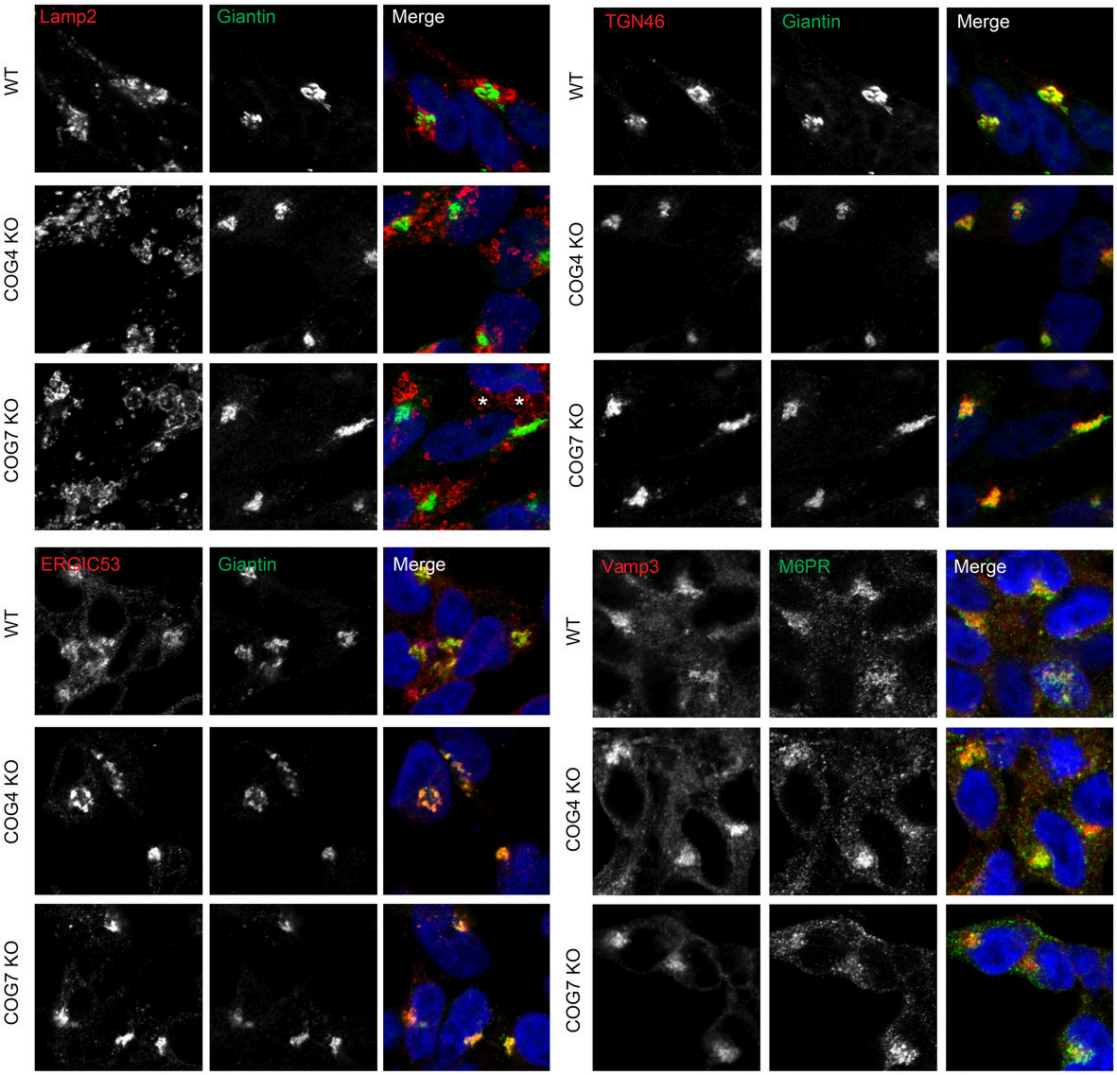
**Golgi and other secretory markers are largely undisturbed in Cog KO cells**. Immunofluorescence of Lamp2, Giantin, ERGIC53, TGN46, Vamp3, and M6PR. Asterisks in Cog7 KO indicate Lamp2-positive, large, endocytic-like structures, Cog4 structures not pictured.

To study Golgi morphology in COG KO cells we subjected cells grown on sapphire disks to high pressure freezing/freeze substitution fixation prior to embedding and sectioning for electron microscopy (EM) analysis. As shown in Figure 4, the Golgi structure is severely distorted in all knock outs compared to control. Golgi cisternae appear dilated and fragmented in all cases, especially in lobe A and in Cog6 KOs (Figure 4, Cog1–4, 6 KOs, arrows). In these cells even Golgi mini-stacks appeared to be disrupted with appearance of multiple autophagosome-like membrane profiles in the Golgi region (Figure 4, arrowheads). In other lobe B KO cell lines strongly dilated Golgi membranes were organized in mini-stacks with the least severe defect observed in Cog5 KO cells (Figure 4, Cog5, 7, 8 KOs, arrows). The morphological changes in the Golgi structure observed in HEK293T KO cell lines appeared to be on par (or possibly even more severe) compared to previously described Cog1 and Cog2 deficient CHO cells (Ungar et al., 2002; Oka et al., 2004; Vasile et al., 2006). Surprisingly this alteration in Golgi structure is only readily apparent via EM. IF analysis of a comprehensive set of Golgi and other secretory protein markers including ERGIC53, GM130, Golgin 84, GalT, p230, Mannose-6-phosphate receptor, Lamp2, and TGN46 in COG4 KO and COG7 KO cells, shows a normal and/or perinuclear distribution, aside from Lamp 2 which localized on large endosomal-like inclusions as well as the normal lysosome distribution (Figure 5 and data not shown).

### Cells deficient in the COG complex show altered toxin binding at the plasma membrane

**Figure 6:**
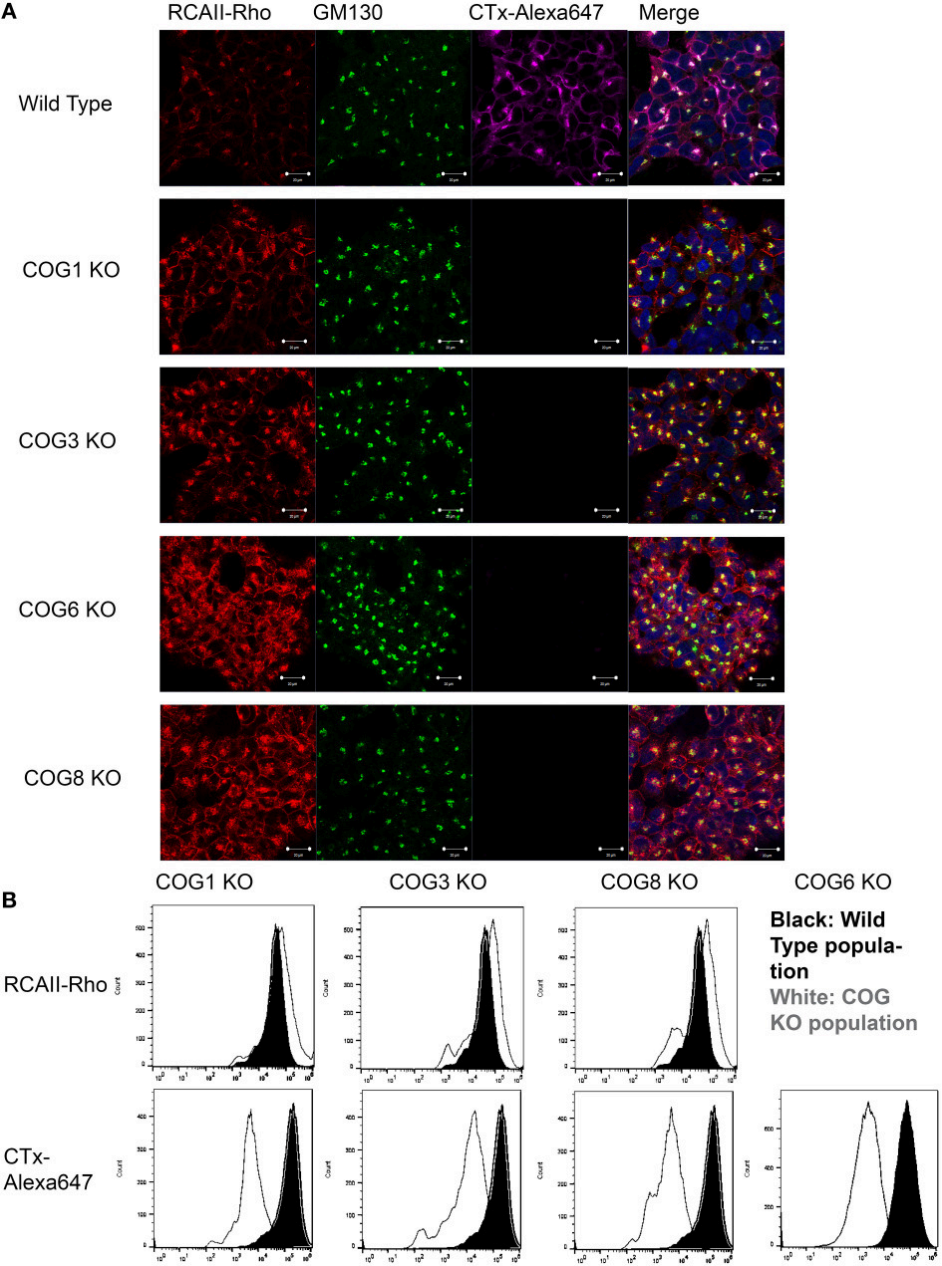
**COG KO cells are altered for the binding of RCA-I and CTxB to plasma membrane**. **(A)** Pulse chase with labeled toxins was done at 37°C for 30 min before fixing and staining with GM130 antibodies. Cells were mounted in DAPI containing media. Coverslips were analyzed using a confocal microscope. **(B)** Cells were incubated with the toxins on ice for 30 min then processed using a flow cytometer and analyzed in FlowJo.

The COG complex is a key player in retrograde trafficking, so we reasoned that intra-Golgi retrograde trafficking should be impaired in cells lacking COG subunits. To test this cell lines were analyzed for their ability to traffic various toxins. Toxins commonly intoxicate a cell by binding to receptors on the plasma membrane then hijacking the hosts' secretory pathway to reach their functional location. For these assays we chose to use one to two KOs for each lobe to represent the lobe as a whole (we felt this was warranted based on KOs destabilizing their lobe partners, Figure 3). The first toxin we attempted to use for retrograde trafficking analysis was Cholera toxin (which is secreted by *Vibrio cholerae* and causes the well-known and deadly effects of Cholera). *V. cholerae* infects cells by utilizing the B subunits of Cholera toxin (CTxB) to bind to glycolipid GM1 at the plasma membrane causing the toxin to be endocytosed (Lencer et al., 1992). Strikingly upon knocking out either lobe A or lobe B COG subunits, binding and endocytosis of CTxB-Alexa 647 in a 30-min pulse chase was almost completely abolished, a phenotype that had not been seen in Cog KD cells. In contrast, wild type cells bind and traffic Cholera toxin to the Golgi after 30 min, visualized by co-localization with GM130 (Figure 6A). The significant reduction in bound CTxB-647 could also be seen by flow cytometry of Cog1, 3 and 8 KO cells compared to wild type populations (Figure 6B). Similarly, it has previously been shown that GM3 levels are significantly reduced in CHO ldlC cells (which are deficient for Cog2). This was attributed to the Cog2 dependent mislocalization of SialT1, which is responsible for the conversion of LacCer to GM3, in these cells (Spessott et al., 2010).

For our next toxin binding and trafficking test, Rhodamine labeled *Ricinus communis* Agglutinin I (RCA-I) was utilized. *R. communis*, or more commonly known as the castor bean plant, produces two kinds of lectins: *R. communis* toxin (RCA-II) (which is well known for its toxicity even at low doses), and RCA-I which is less toxic, but binds galactose residues with high affinity. RCA-1 has been shown to bind tumor cells (which also have altered secretion) with higher affinity than normal cells (You et al., 2010). Interestingly, knocking out COG subunits in either lobe caused a modest increase in RCA-I binding, assessed by fluorescence (Figure 6B). In Cog KO cells RCA-I was also able to traffic to the Golgi after 30 min, though more remained on the plasma membrane than in control cells, indicating a possible delay in retrograde trafficking (Figure 6A).

### Cells deficient in the COG complex subunits have altered intracellular trafficking

**Figure 7:**
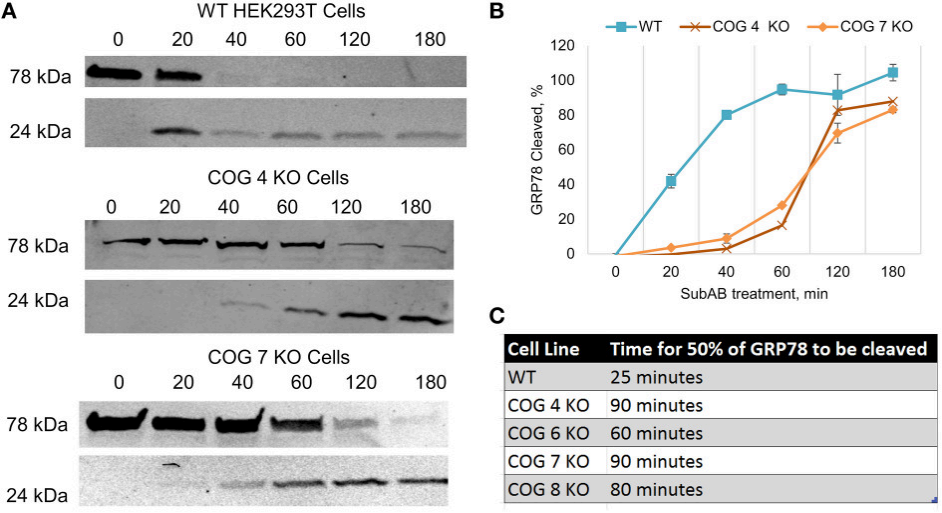
**Retrograde trafficking of SubAB is impaired in COG subunit KO cells**. **(A)** Time course of SubAB-dependent cleavage of GRP78 in control and COG KO cells. Western blot. **(B)** Quantification of GRP78 cleavage. **(C)** Time required for SubAB-dependent 50% cleavage of GRP78 in control and COG KO cells.

Due to the differences in binding of CTXB and RCA-I between wild type and COG KO cell lines that precluded the accurate measuring of retrograde trafficking efficiency of these proteins we turned to the Subtilase cytotoxin (SubAB) trafficking assay (SubAB is a toxin secreted by Shiga-toxigenic *Escherichia coli*). Our group has previously used this assay to study retrograde trafficking in COG knock down cells, and has shown no change in toxin affinity to plasma membrane of COG depleted cells (Smith et al., 2009). SubAB binds at the plasma membrane then is trafficked in a retrograde fashion through the Golgi to the endoplasmic reticulum where it cleaves its target, GRP78 (Glucose-Related protein of 78 kDa). To explore SubAB transport in COG KO cells, these cells were treated with the toxin over time and analyzed via WB for cleavage of GRP78 to smaller fragments (Figure 7A). In all knock out cell lines tested there was a dramatic delay in GRP78 cleavage (Figures 7B,C). Half of GRP78 was cleaved in control HEK293T cells after ~25 min of toxin treatment, but not until ~80 min in COG KO cells (Figures 7B,C). This indicates that retrograde trafficking is impaired in COG KO cells, but not abolished. Significantly, the amount of delay appeared similar in each KO tested regardless of which lobe was affected indicating that the rapid retrograde trafficking of SubAB is dependent on the whole COG complex.

### COG deficient cells missort cathepsin D

**Figure 8:**
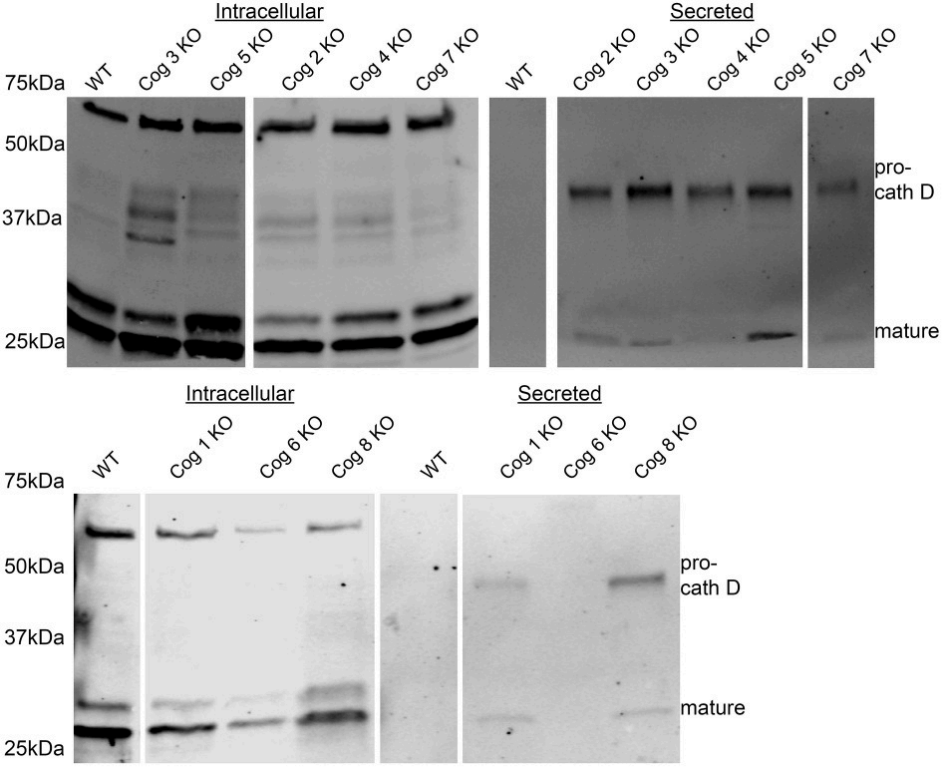
**Pro-cathepsin D is secreted from COG subunit KO cells but not WT cells**. Cells at 80–100% confluency were placed in chemically defined serum free media. Media was collected at 24 h and then analyzed by western blot for Cathepsin D.

Next we probed the COG KO cells lacking lobe A or B subunits for other possible trafficking defects by analyzing conditioned serum-free, chemically-defined culture media taken after 48 h of incubation with cells. We found that all cells lacking COG subunits (with the exception of Cog6) but not the original HEK293T cells secrete immature Cathepsin D (a lysosomal protease), indicating defects in post-Golgi sorting and/or endosome/lysosome impairments (Figure 8). Interestingly some, but not all KOs also secrete a small amount of mature Cathepsin D. Because Cog6 KO was the only outlier for immature Cathepsin D secretion we tested another Cog6 KO clone which yielded a similar result (data not shown), though further analysis of this cell line and generation and analysis of more Cog6 KO cell lines will be needed in the future to confirm this finding.

### Cells deficient in the COG complex show misglycosylated Lamp2, reduced sialylation and fucosylation, and changes in oligomannose composition

**Figure 9:**
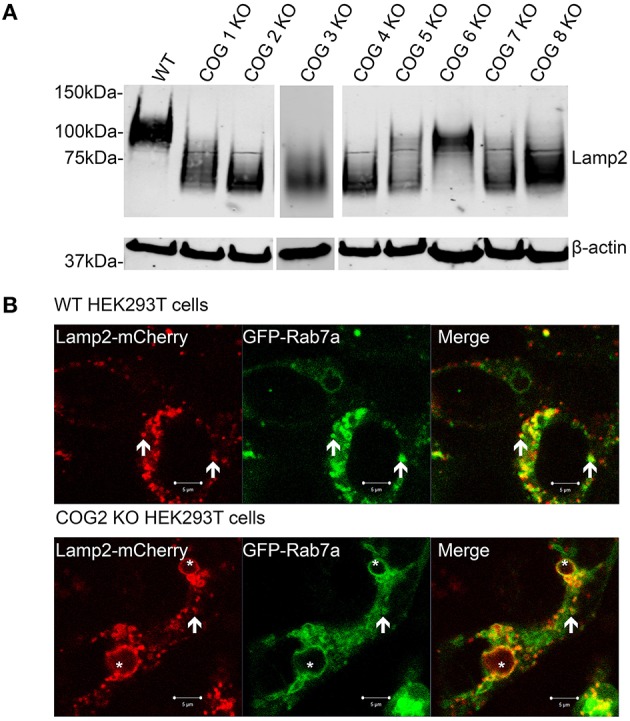
**Lamp2 is hypoglycosylated in COG subunit KO cells, but still localizes to lysosome like structures**. **(A)** Cell lysates were analyzed by western blot for Lamp2 mobility. **(B)** WT and COG 2 KO cells were transfected with Lamp2-mCherry and GFP-Rab7a. Arrows represent colocalization. Asterisks represent large, late endocytic inclusions. Images taken of live cells.

Increases in GNL binding to plasma membrane proteins, along with mistargeting of Cathepsin D indicate potential severe defects in cellular lysosomal machinery and specifically in lysosomal Lamp2 protein. Earlier we had noted Lamp 2 labeling large endosomal like inclusions by IF in COG KO cells from lobe A and B, so we decided to investigate this further. Lamp2 is a heavily glycosylated protein (10 O-linked and 16 N-linked glycosylation sites, most of which are utilized), and is primarily located on lysosomal membranes. It is thought to help in lysosomal biogenesis and dynamics (Eskelinen et al., 2002; Saftig and Klumperman, 2009). We used western blotting to assess the glycosylation status of Lamp2 by assessing shifts in the protein's mobility. We have previously observed that Lamp2 electrophoretic mobility is increased in cells treated with siRNA to Cog3 and Cog7 (Shestakova et al., 2006). Indeed, in cells lacking COG complex subunits Lamp2 had a greatly increased mobility. This difference was not lobe specific, though the absence of some subunits appears to cause a greater increase in mobility than others (2, 3, 4, and 7 had the highest mobility; Figure 9A). At the same time the majority of hypoglycosylated Lamp2 was still properly localized to lysosome-like intracellular structures as determined by colocalization with Rab7a, indicating, indicating the trafficking of transmembrane proteins and general lysosomal biogenesis is mostly undisturbed in COG KO cells (Figure 9B).

**Figure 10:**
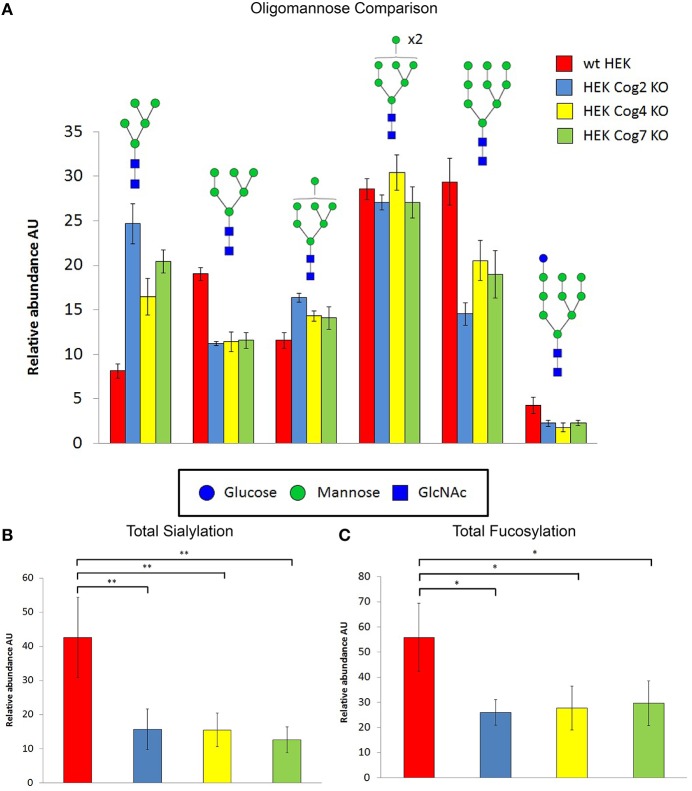
**COG KO cells have global changes in *N*-linked glycans structures**. *N*-glycans prepared from cell lysates were profiled by mass spectrometry. The normalized intensities for individual glycans grouped into different classes as indicated were summed. Error bars show SEM for *n* = 4 (WT, Cog2KO), *n* = 5 (Cog4KO), *n* = 8 (Cog7 KO). **(A)** Oligomannose composition changes in KO cells vs. WT cells. **(B)** Sialyation changes in KO cells vs. WT cells. ^**^denotes *p* < 0.01. **(C)** Fucosylation changes in KO cell vs. WT cells ^*^denotes *p* < 0.05. For **(B,C)** one-way ANOVA with a Sidak-Holm *post hoc* statistical significance test was utilized.

To gain a better understanding of the changes in glycosylation upon loss of individual COG subunits we further analyzed COG KO cell lysates by using filter aided *N*-glycan Separation (Abdul Rahman et al., 2014) followed by mass spectrometric *N*-glycan profiling. For this analysis we chose Cog4 and 7 KOs to represent the 2 lobes (we later chose to analyze Cog2 KO as well to confirm the latter glycosylation impairments that were originally thought to only occur in lobe B mutants). Profiling revealed numerous glycosylation changes, the most prominent of which are summarized in Figure 10. As observed previously for the Cog1 and Cog2 deficient ldlB and ldlC CHO cell lines (Abdul Rahman et al., 2014) the oligomannose distribution shifts in the mutants, with a significant increase in the Man_5_GlcNAc_2_ species and a concomitant decrease in the level of the Man_6_GlcNAc_2_ glycan (Figure 10A). The observed increase in the Man_5_ glycan fits well with the increased GNL affinity of these cells, since this glycan species has two terminal 1–3 linked mannose residues (Shibuya et al., 1988). In addition to the increase in Man_5_ glycan there is also a significant decrease in the Man_9_GlcNAc_2_ glycan's abundance, which was not observed in the CHO mutants. Other functionally important changes are overall decreases in the levels of sialylation (Figure 10B) and fucosylation (Figure 10C) in all KO mutants analyzed. These are changes that often affect the binding of important ligands to cell surface glycans. A decrease in protein sialylation has been observed before in CDG-II patient fibroblasts (Wu et al., 2004; Kranz et al., 2007; Zeevaert et al., 2008), and defects in fucosylation have been seen in *C. elegans* Cog1 mutant (Struwe and Reinhold, 2012). Interestingly, all observed glycosylation changes, no matter if dependent on early-, medial,- or late-Golgi processing steps, were very similar for Lobe A (Cog2 and 4) and Lobe B (Cog7) knockouts, indicating that the whole complex is needed for proper glycan processing to take place.

## Discussion

As a result of this work a complete set of HEK293T cell lines void of individual COG subunits was created. These cells are an improvement on past KD and mutant studies as they are all in the same cell type. We believe that this set will provide the unique opportunity not only in the ongoing investigation of the specific function of each individual subunit of this multifunctional MTC but also as a starting reference point for detailed and unbiased characterization of the other MTCs. Each COG KO cell line can also be used to characterize (and potentially fix) glycosylation and trafficking defects associated with naturally occurring mutations in COG complex subunits that cause CDGII and other human maladies.

Thus far all COG subunit knockouts have been shown to have: altered *G. nivalus* lectin (GNL) binding to plasma membrane glycoconjugates, altered binding and trafficking of toxins, *cis*, medial, and *trans*-Golgi glycosylation defects, altered Golgi morphology, and dramatic fragmentation of the trans Golgi network, altered Cathepsin D secretion, and hypoglycosylated Lamp2.

As mentioned above, in ldlC (Cog2 deficient) CHO cells it has previously been recognized that GM3 synthesis is impaired (Spessott et al., 2010). GM3 is a precursor to GM1 (the binding site for Cholera toxin) in the a-series ganglioside synthesis pathway (Kolter et al., 2002). Spessott et al attributed this reduction in GM3 synthesis to COG dependent mislocalization of SialT1. Our COG KO cells show a global deficiency in sialylation, making a similar reduction GM1 the most likely explanation for our drastic reduction of Cholera toxin binding in our human COG subunit deficient cells, but that this deserves further investigation.

*G. nivalus* lectin (GNL) binding is known to be increased in COG KD cells (Shestakova et al., 2006; Richardson et al., 2009; Pokrovskaya et al., 2011; Willett R. A. et al., 2013; Ha et al., 2014). We have shown that this increased lectin affinity is also seen in CRISPR created COG KO populations (with perhaps even a higher affinity than the COG KDs) and is sufficient for effective FACS analysis and sorting to create clonal COG KO populations. GNL binding shows a similar affinity for all of the COG subunit KOs indicating substantial defects in cis/medial-Golgi glycosylation when either lobe of the COG complex is impaired. This contradicts the previous COG complex models which suggest lobe A but not lobe B having a primary role in *cis*/medial glycosylation (Reynders et al., 2009; Peanne et al., 2011). Mass spectrometric analysis of the *N*-linked glycan revealed further similarities in glycosylation defects between cells deficient in all COG subunits tested so far (2,4,7), differing from previous suggestions that lobe B is primarily responsible only for the latter stages of glycosylation (Peanne et al., 2011; Laufman et al., 2013). We have observed that each of our COG KO cell lines have the same characteristic alteration in their oligomannose distributions, as well as impaired sialylation and fucosylation.

Due to observed glycosylation changes, we decided to analyze a panel of Golgi and other secretory markers; GM130 (a rod-like protein with coiled-coil domains located in the cis-Golgi), TGN46 (a protein localized to the trans-Golgi network), ERGIC53 (a mannose specific lectin carrier in the ER-Golgi intermediate compartment), Giantin, Lamp2, and Mannose-6 phosphate receptors were analyzed by immunofluorescence. We observed no significant effect on localization of tested Golgi or other secretory markers in COG KO cells at the IF level, aside from large Lamp2 positive endocytic like inclusions that were observed in addition to normal lysosomal Lamp2 distribution. Further observations revealed that Lamp2 is also hypoglycosylated in COG KO cells. Though properly localized aside for the endocytic inclusions, this hypoglycosylation could result in altered lysosomal function or trafficking and will be an interesting area of future investigation.

Following IF, more precise analysis of Golgi structure using EM revealed that knocking out COG complex subunits causes extensive dilation and fragmentation of the Golgi. Though all subunits appear to be important for maintaining normal Golgi structure, the level of Golgi dispersion is subunit dependent with the lobe A KOs and Cog6 KO having the most disrupted Golgi structure, indicating certain COG subunit interactions may be more important for Golgi stacking than others.

We have observed Cog3, 4, 5, 6, 7, and 8 subunit levels in each of the COG KOs. Lobe A KOs affect Cog3 and Cog4 levels (Cog2, 3, and 4 KOs dramatically affected these, while Cog1 KO has much less impact). Lobe B KOs have little/no effect on lobe A. Cog6 and 8, protein levels also affect their lobe partners the most with Cog6 being most affected in Cog5, 6, and 7, and Cog8 being most affected in Cog5 and 6 with Cog1 and Cog7 KOs being moderately affected. This Cog1 KO and Cog8 protein effect is expected and gives further support to the Cog1-Cog8 bridging interaction (Ungar et al., 2005), which could also lend stability to each of the proteins *in vivo*. Importantly, the dramatic lobe specific effects on protein stability do not carry over to the opposing lobe indicating that the similarity in phenotypes between lobe A and lobe B subunit KOs is not due to instability of the complex, but rather to the critical function of each lobe in glycosylation and Golgi structure.

In line with impaired lysosomal trafficking and/or function indicated by lamp2 hypoglycosylation, immature Cathepsin D secretion was observed in COG complex subunit KOs. Cathepsin D is a lysosomal aspartic protease that undergoes a conformational change in the lysosome to become catalytically active. Its inactive form has also been shown to have a role in apoptosis. Procathepsin D is also secreted from cancer cells where it has a mitogenic effect on surrounding cells (Benes et al., 2008). These results indicate that loss of COG function could affect other aspects of the secretory pathway indirectly and change the way organelles and cells function in their environment. To this effect, we have also observed large intracellular accumulations in the COG KO cells that appear to be of endocytic/phagocytic origin. These accumulations appear mostly void of electron dense material and, in the most severe cases take up ~50% of the cells' volume (Blackburn and Lupashin, data not shown).

In the future, these COG KO induced accumulations, and how other subunit specific interactions (with SNAREs, Rabs, and other tethers) are affected in COG complex subunit KO cells will be an important area of investigation. Improper Golgi structure and function is a hallmark of human diseases ranging from cancers to Alzheimer's. This makes further understanding the key players of the secretory pathway, such as the COG complex essential for human health.

## Author contributions

JB wrote the article and made substantial contributions to conception and design, acquisition of data, analysis, and interpretation of data. IP participated in drafting the article, performed electron microscopy, and interpreted the data. PF participated in drafting the article, performed glycan analysis, and interpreted the data. DU participated in drafting the article, performed glycan analysis, and interpreted the data. VL wrote the article and made substantial contributions to conception and design, acquisition of data, analysis and interpretation of data.

### Conflict of interest statement

The authors declare that the research was conducted in the absence of any commercial or financial relationships that could be construed as a potential conflict of interest.
